# The Correlation between Nurses’ COVID-19 Infections and Their Emotional State and Work Conditions during the SARS-CoV-2 Pandemic

**DOI:** 10.3390/ijerph182312715

**Published:** 2021-12-02

**Authors:** Iwona Malinowska-Lipień, Magdalena Suder, Tadeusz Wadas, Teresa Gabryś, Maria Kózka, Agnieszka Gniadek, Tomasz Brzostek

**Affiliations:** 1 Institute of Nursing and Midwifery, Faculty of Health Sciences, Jagiellonian University Medical College, 31-501 Kraków, Poland; imalin@poczta.onet.pl (M.S.); ter.gabrys@uj.edu.pl (T.G.); maria.kozka@uj.edu.pl (M.K.); agnieszka.gniadek@uj.edu.pl (A.G.); tomasz.brzostek@uj.edu.pl (T.B.); 2 Małopolska District Chamber of Nurses and Midwives in Krakow, 31-501 Kraków, Poland; twadas@moipip.org.pl

**Keywords:** emotions, nurses, COVID-19, workplace, workload

## Abstract

The COVID-19 pandemic caused by the SARS-CoV-2 virus has significantly influenced the functioning of Polish hospitals, and thus, the working conditions of nurses. Research on the presence of specific negative emotions in nurses may help identify deficits in the future, as well as directing preventive actions. The present research was performed among nurses (*n* = 158) working in Polish healthcare facilities during the third wave of the COVID-19 pandemic caused by the SARS-CoV-2 virus, where Group A (*n* = 79) consisted of nurses diagnosed with COVID-19, and Group B (*n* = 79) nurses who have never been infected with COVID-19. To perform the research, the Courtauld Emotional Control Scale (CECS), Trait Anxiety Scale (Polish: SL-C) and the authors’ survey questionnaire were used. A positive test result was generally determined more often among nurses who indicated a noninfectious ward as their main workplace, compared to nurses employed in infectious wards (64.55% positive vs 33.45% negative). Over a half of the subjects identified moderate levels of emotion suppression as the method to regulate strong emotions, while one-quarter cited high levels of suppression. Anxiety was suppressed at high and moderate levels by 97% of the subjects, depression by 86.71%, and anger by 79.48%. Infection with COVID-19 results in a higher level of anxiety and depression, as well as a feeling of increased work load.

## 1. Introduction

The appearance at the end of 2019 and quick spread throughout the world of the SARS-CoV-2 virus causing the COVID-19 led to the announcement of a pandemic by the World Health Organisation (WHO) and the introduction of guidelines for epidemiological activities [1]. The most frequent mode of SARS-CoV-2 transmission is via respiratory droplets [2]. Clinical imaging of SARS-CoV-2 infection varies from asymptomatic infection to acute respiratory failure, and in extreme cases, multiple organ failure or even death [3]. Underlying diseases and age significantly influence the course of the infection, increasing the risk of death [4]. Typical symptoms are elevated temperature 38.5–39.0 °C, cough—usually dry, acute dyspnoea and pain in the chest. Accompanying symptoms are smell and taste disorders as well as conjunctivitis and gastrointestinal issues [1].

Studies have shown that COVID-19, besides leading to somatic changes, may also cause psychic ones, from mild to acute psychiatric symptoms [5,6]. Social distancing and isolation may trigger numerous abnormal reactions, manifested by post-traumatic stress, anger and a sense of loss [7,8,9]. The pandemic has caused disturbances in everyday routines of human life [10]. People all over the world are troubled by uncertainty, job losses, isolation and separation from their families, as well as fear of getting infected and death. Serious effects on the psychological and physical condition of medical staff have already identified in previous pandemics [2,3,11]. Undoubtedly, physicians, nurses and paramedics working directly with patients suspected of harboring infection perceive a significant threat to their well-being. A serious burden for medical personnel has been awareness of the risk of becoming infected, and then going to on infect their families. In fighting the pandemic, many medical workers have themselves become infected, and some died. Since the beginning of the pandemic in Poland, 2,923,304 cases of SARS-CoV-2 infection have been confirmed, of which 57,060 concerned nurses. The records of the Ministry of Health show that 185 nurses have died in Poland from the beginning of the pandemic to October 2021 [12,13]. In addition to the growing mortality related to SARS-CoV-2 infection, medical workers have been subjected to longer work hours, more difficult work conditions and increased workloads through the imposition of additional activities [14,15]. Fear resulting from the COVID-19 pandemic potentially influences efficiency and workplace safety. Additionally, long-term negative emotions increase the risk of somatic ailments. What is essential is immediate and long-term support for personnel based on analyses of the influence of negative emotions on health [7,16,17]. 

Our research is among the first to tackle the issue of emotions and control over them in a group of working nurses, including those infected with COVID-19. The results of the present research may aid healthcare management to undertake actions to reduce the effects of the pandemic on healthcare employees. 

The aim of the present work was to determine the relation between COVID-19 infections among nursing staff and emotion levels, as well as to evaluate changes to work conditions during the SARS-CoV-2 pandemic. 

## 2. Materials and Methods

### 2.1. Study Design

This research used the diagnostic poll method, implementing the Courtauld Emotional Control Scale (CECS) to collect data [18], as well as the Trait Anxiety Scale (Polish acronym: SL-C) [19] and the authors’ own survey questionnaire. The Emotional Control Scale CECS was developed by Watson M. and Greer S., and was adapted for a Polish setting by Juczyński. [18]. The scale is aimed at measuring the ability to control subjective emotions in difficult situations. It consists of three subscales: (1) anger control, (2) anxiety control, and (3) depression control. The reliability (Cronbach’s alpha) of the Polish version of the CECS scale reached 0.80 for anger control, 0.77 for depression control,0.78 for anxiety control, and 0.87 for the general emotion control coefficient (CECS) [18]. Each of the subscales contains seven statements, and answers are provided according to a four-point scale, from “hardly ever” (1 point) to “almost always” (4 points). The sum of the points constitutes the emotion control coefficient for the particular subscale, whose range for each is from 7 to 28 points. The sum of the received results from the three subscales presents the general coefficient of emotion control (CECS), which is within a range of 21–84 points. The core of the present research is the quantification of a subjective value describing the ability to control reactions while experiencing negative emotions. Higher scores indicate more pronounced suppression of negative emotions. According to the CECS scale grading, three levels of emotional suppression for the general emotion control coefficient (CECS) were accepted for the analysis: low (<42 points), medium (from 42 to 63 points) and high (>63 points); and respectively for subscales anger, anxiety, depression: low (<14 points), medium (14 to 21 points) and high (>21 points) [18]. The second tool used in our research was the Trait Anxiety Scale (Polish: SL-C), a standardized tool developed by M. Piksa et al. which serves to evaluate anxiety level as a personality trait. The reliability (Cronbach’s alpha) of the Polish version of the SL-C scale reached 0.86 [19]. The questionnaire consisted of 15 questions. The answers were given in a four-grade scale, from often—3, sometimes—2, rarely—1, and never—0. Before calculating the total, a change is made by reversing the grading of statements 9 and 11. The sum of all points is the general coefficient of anxiety trait intensity, represented by a value between 0 (minimal intensity) and 45 (maximum intensity) (19). The author’s poll questionnaire consisted of 54 questions concerning work conditions during the pandemic, as well as COVID-19 infection and a series of questions concerning socio-demographic variables (age, gender, education, work experience and workplace). 

### 2.2. Participants

Complete responses were obtained from 162 nurses. Four were excluded from our analyses because the respondents did not indicate that they consented to participate in the study. The poll questionnaire was filled out by 158 nurses, all of whom gave their consent to participate in the study. Subjects were divided by prior COVID-19 infection confirmed by a PCR test, i.e., 79 nurses who were presently or had been infected with COVID-19 (Group A), and 79 healthy nurses who had never had COVID-19 (Group B). 

### 2.3. Data Collection

The research was carried out between December 2020 and March 2021 on a group of nurses (male/female) working in the southern regions of Poland. It was a part of a larger project seeking to valorize the roles of nurses during the SARS-CoV-2 pandemic, performed by Malopolska District Chamber of Nurses and Midwives in Krakow (Polish: MOIPiP) [20]. The trial was goal-oriented. Research was done by means of an online poll, which was only available for nurses. Participation in the study was voluntary and with a guarantee of anonymity. In December 2020, a link to the poll questionnaire was sent by email to 74 representatives of MOIPiP, i.e., nurses actively supporting the professional organization. Participants were encouraged to forward the questionnaire link to their nurse colleagues who met the criteria for participation in the research, using the snowball sampling method. The criteria of inclusion in the research were: (1) consent to participate; and (2) actively working in nursing/performing duties during the pandemic, at least since March 2020 (i.e., from the moment of the announcement of a pandemic in Poland). The exclusion criteria were: (1) lack of consent to participate in the poll; (2) no active work in the nursing profession (e.g., retirement, disability, inability to work, suspension of the right to work, parental leave, etc.); and (3) termination of professional duties before March 2020, i.e., before the pandemic in Poland. 

### 2.4. Ethical Procedures

The research was performed according to the ethics rules of scientific research involving humans as laid out in the Declaration of Helsinki, and was voluntary and anonymous. Permission to undertake the research described above was obtained from the Bioethical Commission of Jagiellonian University Medical College in Krakow (No. 1072.6120.346.2020, 16 December 2020). 

### 2.5. Statistical Analysis

A statistical analysis was performed using the SPSS Statistics software (IBM, Armonk, NY, USA). To describe the quantitative variables and the structure of the researched group, numbers (*n*) and percentages (%) of persons showing a certain feature were used. The description of measurable variables was presented using descriptive statistics values (mean value (*x*), minimum (Min), maximum (Max), standard deviation (SD)). To determine compliance with the normal spread, the Kołmogorow-Smirnow test was used (D). Correlations among quantitative variables were verified using the Pearson correlation coefficient (PCC) (r). For the statistical analysis, to determine the influence of independent variables on emotion control level and anxiety intensity as personality traits, the Kruskal-Wallis test was used (H), enabling us to determine differences between a large number of independent samples. To verify the dependence or lack thereof of particular variables, the independence χ^2^ (chi-squared) test was used, which allowed us to compute the significance for more than two differences between the groups. For the 2 × 2 tables, the statistics of the χ^2^ test with Yates’s correction was calculated. In all analyses, a significance level of *p* ≤ 0.05 was accepted, indicating the presence of statistically significant dependencies. 

## 3. Results

The researched group consisted of 158 people, comprising 152 women (96.2%) and 6 men (3.8%). The age of the studied subjects ranged from 23 to 63 years. The average age was 36.8 ± 10.91 years. The most numerous were aged from 26 to 35 years (*n* = 50; 31.65%). The smallest group comprised persons over 55 years of age (*n* = 8; 5.06%). The majority of the analyzed group were married (*n* = 88; 55.7%). Over half of the subjects declared holding a master degree in nursing (*n* = 89; 56.33%). Work experience in the group was as follows: one-third of them cited between 1 and 5 years (*n* = 57; 36.08%) or experience, while one-fifth had been working for 21 to 30 years. Most of the subjects (*n* = 104, 65.83%) were employed in noninfectious hospital wards; see Table 1. 

Among the research subjects, half (*n* = 79) had experience COVID-19 infection, confirmed by a positive PCR (Polymerase Chain Reaction) test for SARS-CoV-2 infection (Group A). The other half had never been infected with COVID-19 (Group B). SARS-CoV-2 infections occurred most frequenting in the autumn of 2020, namely during the second wave of the pandemic. Among the Group A studies cases, one quarter (*n* = 40) fell ill from September to October 2020 (25.32%), and one fifth (*n* = 34) in November and December 2020 (21.52%). In the initial phase of the COVID-19 pandemic, i.e., in the spring of 2020, five persons became infected (3.16%). While the research was being performed, six nurses became infected with COVID-19 and remained in isolation. In Group A, a dominating symptom of infection was asthenia, which was reported by 72 nurses (91.14%), followed by headache (*n* = 61, i.e., 77.22%), loss of smell (*n* = 57, i.e., 72.15%), and muscle pains (n = 55, i.e., 69.62%). Two thirds of the researched cases indicated having experience a loss of taste (*n* = 49; 62.03%), and almost half experienced coughing (*n* = 38, i.e., 48.10%). Low-grade fever occurred in 40.51% of the study subjects (*n* = 32), and 22.78% experienced a high temperature, i.e., >38°C (*n* = 18). One quarter of the subjects experienced dyspnea (*n* = 20; 25.32%). Diarrhea was reported by 24.05% (*n* = 19) of subjects. A lack of any symptoms despite a positive SARS-CoV-2 test was indicated by one person (1.27%); see Table 2.

In Group A, almost two-thirds (*n* = 49, 62.03%) assessed their health as good prior to becoming infected, one-quarter (*n* = 21, 26.58%) as bad, a one in ten (*n* = 9, 11.39%) as very bad. Most of the polled nurses from both groups (A and B), i.e., two-thirds (*n* = 106, 67.09%), indicated that no SARS-CoV-2 infection had been detected among their household members, while one-third (*n* = 52, 32.91%) cited the contrary. The presence of the SARS-CoV-2 virus among family and household members was confirmed much more frequently in Group A than Group B (49.37% vs.16.46); *p* = 0.00002. 

### 3.1. Analysis of Emotions Experienced by Nurses while Working during the SARS-CoV-2 Pandemic

The pandemic caused increased anxiety among nurses, as declared by 44.31% (*n* = 70;) of subjects from both groups. Over a half of the polled nurses noted having experienced mild anxiety (*n* = 84; 53.16%), while a lack of anxiety related to the pandemic was reported only by 3.16% (*n* = 5). A higher percentage of individuals from Group B experienced strong anxiety compared to Group A (15.19% vs 8.86%); *p* = 0.56. Because of the pandemic, most of the subjects from both groups (*n* = 146; 92.41) experienced increased stress levels associated with their work; only eight subjects expressed the opposite opinion (5.06%), and four persons were not able to estimate changes in their stress levels (2.53%). In Group A, 94.94% declared the perception of increased stress level due to the pandemic; in Group B, this value was 89.87% (*p* = 0.45). In the group of quarantined nurses, isolation had a negative influence on their mental condition, as confirmed by 76.27% of subjects (*n* = 55). A significantly more frequent negative influence of quarantine on mental condition was indicated by the nurses from Group A compared to Group B (50% positive vs 27.59% negative), *p* = 0.02. 

In the both groups, the values on the emotion control scale (CECS) were within a range from 25 to 81 points; the mean result of this scale was 54.7 ± 11.53. The highest mean value was indicated in the anxiety subscale, i.e., 18.31 ± 4.42, followed by the depression subscale 18.25 ± 4.43. The lowest result was recorded in the anger subscale, i.e., 18.14 ± 5.05. 

**Figure 1:**
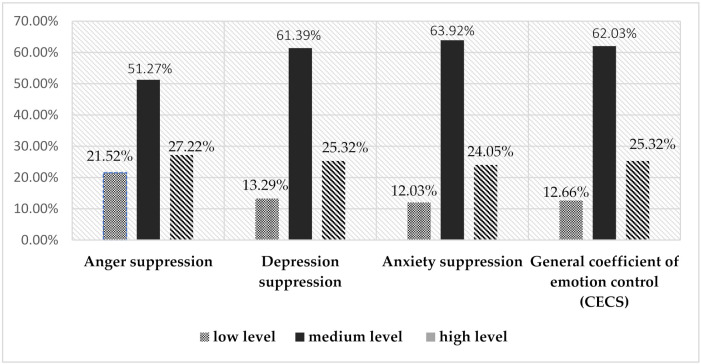
Percentage spread of emotion suppression levels according to the CECS scale.

Over a half of the study subjects noted medium level emotional suppression as a method to manage strong emotions, while one-quarter cited high levels. Anxiety was suppressed at high and medium levels by 87.97% of study subjects, depression by 86.71%, and anger by 79.48% (Figure 1). 

Our analysis of anxiety level as a personality trait, i.e., compared to the constant value present in human life according to the SL-C scale, showed that the coefficient of anxiety intensity in the research group ranged from 2 to 44 points. The mean value of this scale was 25.35 ± 7.97, with 26.53 in Group A and 24.18 in Group B; *p* = 0.04. The result of SARS-Cov-2 testing was not an important factor influencing the levels of emotional suppression. However, nurses from Group A showed a higher anxiety intensity compared to those in Group B (*p* = 0.04); Table 3.

The anxiety intensity scale (according to SL-C) was correlated with the depression subscale (according to CECS); this correlation was particularly strong in Group A (*p* = 0.01), Table 4.

### 3.2. Analysis of Work Conditions during the SARS-CoV-2 Pandemic

A positive SARS-CoV-2 test result was more frequent in nurses working in noninfectious wards of hospitals compared to those working in infectious wards (64.55% vs. 33.45%, *p* = 0.0008). Most subjects from both groups had contact with patients suspected of SARS-CoV-2 infection as part of their work (*n* = 57, i.e., 36.08%), either “often” or “very often” (*n* = 56, i.e., 35.44%). Most of the nurses confirmed that in their workplace, procedures had been implemented regarding dealing with SARS-CoV-2 infected or potentially infected patients (*n* = 148; 93.67%). In the group which claimed that no such procedures had been implemented, positive SARS-CoV-2 test results were obtained twice as frequently (5.06% positive vs. 2.53% negative); *p* = 0.09. According to 56.96% (*n* = 90) of the study subjects, training sessions had been organized in the workplace concerning the use of personal protective equipment (PPE) (i.e., putting on/taking off protective clothing, such as overalls). One third of the polled nurses stated that they had to educate themselves on their own (*n* = 57; 36.08%). Additionally, 6.96% (*n* = 11) of nurses had no knowledge on this topic. In the polled group which stated that no training sessions had been provided in their workplace or they had no knowledge on the topic, positive results for SARS-CoV-2 test were obtained substantially more frequently (49.37% vs. 36.71%, *p* = 0.014). Out of the entire group, most claimed that they were working in wards equipped with sufficient personal protective equipment (*n* = 110; 69.62%), and were given adequate access to such equipment (*n* = 111; 70.25%); *p* > 0,05. The possibility of undergoing a control swab in the workplace to test for SARS-CoV-2 infection was declared by 50.63% (*n* = 80) of subjects. One in five subjects (*n* = 35; 22.15%) claimed that they had to apply to do a test in the workplace, while 43 subjects had no possibility of doing a COVID-19 test in the workplace, among whom 10.13% cited a lack of control swab acquisition points (*n* = 16), and 17.09% a lack of consent from their employer (*n* = 27), as the reasons. The option of doing a COVID-19 test in the workplace was much more widely indicated by nurses in Group B compared to Group A (58.23% vs. 43.04 %; *p* = 0.00005). Nurses in Group A, more often than those in Group B, were unable to undergo control tests due to a lack of employer consent (29.11% positive vs. 5.06% negative); *p* < 0.05. Meanwhile, 56.9% (*n* = 90) of the study subjects could rely on their employer’s and supervisors’ support in this regard. Support from supervisors was statistically more often declared by nurses from Group B than Group A (67.09% negative vs. 46.84% positive); *p* = 0.02. 

The support of a psychologist in the workplace was cited by only one-quarter (*n* = 40, 25.32%) of the study subjects; all others (*n* = 118; 74.68%) had no such possibility. Group A, substantially more frequently than Group B, cited an absence of professional psychological aid (84.81% with a positive result vs 64.56% with a negative result, *p* = 0.006). Most of the polled nurses (*n* = 152; 96.2%) could count on the support of the coworkers. During the pandemic, the perception of increased workload in the workplace was declared by 84.18% (*n* = 133) of nurses. Increased workload was indicated much more often by the nurses from Group A than Group B (89.87% vs. 78.48%); *p* = 0.04. Most of the study subjects observed shortages of medical personnel in the workplace (*n* = 135; 85.44%). During the pandemic, 57.59% worked in the 12-h shift system (*n* = 91), 16.46% in the alternate 12-and-24-h system (*n* = 26), and 13.29% in the 24-h shift system (*n* = 21). Due to the pandemic, 68.99% of the polled nurses (*n* = 109) worked overtime, i.e., 70.89% from Group A and 67.09% from Group B; *p* = 0.73; Table 5.

## 4. Discussion

The nursing profession requires mental resistance and the ability to cope with difficult situations. The current situation falls into such a category. For medical professionals, working in pandemic conditions takes not only a physical toll, but also a mental one. New and unknown situations, as well as feelings of danger, lead to the appearance or escalation of negative emotions, such as anger, anxiety or aggression [15]. 

The present research demonstrated that the SARS-CoV-2 pandemic has negatively influenced nurses’ emotions, leading to high levels of stress and anxiety among the majority of them. In research by Handy et al., moderate and high levels of stress were reported by 78.3% of nurses working in hospitals during the pandemic. That research revealed that fear of infection or of infecting family members had a substantial influence on stress levels, as did a lack of access to personal protective equipment and training sessions related to COVID-19 [21].

The present research concludes that the vast majority of nurses use the emotion suppression mechanism as a way of coping with negative emotions related to the SARS-CoV-2 pandemic. This applies in particular to the suppression of anxiety, which was indicated by 87.97% of subjects, followed by depression in 85.71%, and anger in 79.48%. In research performed in 285 hospitals throughout Poland on, among other things, the work conditions experienced by medical personnel during the COVID-19 pandemic, Buchelt B et al. demonstrated that the pandemic has adversely affected the mental condition of as many as 38% of nursing staff. The most commonly-experienced emotions were anxiety, high levels of stress, fear, helplessness, anger and fear for one’s own health and life, as well as those of relatives. Work with patients infected or suspected being infected with SARS-CoV-2 was a major factor behind the increase in perceived negative emotions [22]. Another study performed before the pandemic demonstrated that nurses, in general, regularly experience strong anxiety. This may result from the fact that the core of their work is related to responsibility for human life. Anxiety as a trait may be an inherent or acquired behavioral inclination maintained throughout one’s life. A high intensity of this trait influences the perception of potentially nonthreatening situations as threatening ones, which, in turn, trigger anxiety reactions which are disproportionate to the situation [23]. The current epidemiological situation has had an impact on the anxiety levels experienced by the majority of nurses, as shown in our research and elsewhere [24]. In difficult situation which trigger negative emotions, the ability to control them constitutes the fundamental mechanism for coping with stress. A real ability to control emotions is demonstrated by the possibility to react appropriately when faced with a threatening situation. Research by Mocan et al. focusing on nurses working during the COVID-19 pandemic demonstrated that persons experiencing higher than usual levels of anger and rage when faced with difficult situations frequently chose strategies based on emotions. The perception of fear, in turn, was correlated with focusing on the problem and task, and not on the suppression of emotions such as sadness or anger [23]. Emotional control and coping with stress may be enhanced by a certain level of phase ability related to the pandemic and the acquisition of knowledge thereof. Experiencing sensations of shock, fear and chaos led many individuals to inform themselves, create procedures and acquire experiences as constructive preventive and therapeutic actions [23]. In 2019 and 2020, nurses faced a previously unknown infection, one which they were initially deprived of effective treatment methods to prevent. The increasing number of infections, deaths and an atmosphere of panic in society at large evoked anxiety and fear, the levels of which were definitely higher than under normal circumstances. Prior potential biological threats, e.g., SARS, MERS, or even the AH1N1 influenza, did not spread to nearly the same extent as the current pandemic, either in Poland or elsewhere. Nurses working during the SARS-CoV-2 pandemic experience fear related to the risk of infecting themselves and their relatives [25]. Many nurses made the decision to temporarily stay in hotels, as the desire to protect family members was stronger than the need for support provided by loved ones [26]. 

Important factors conditioning the emotions perceived by nurses and their ability to control them are the type of ward in which they work and the overall work conditions. Among the study subjects, nurses employed in noninfectious hospital wards showed a higher rate of SARS-CoV-2 infection than those working in infectious wards. At the same time, in the group which cited a positive SARS-CoV-2 test result, the intensity of fear and depression suppression were significantly higher, as was the perception of an increase work load. 

During the COVID-19 pandemic, most of the study subjects observed deficiencies in the workplace, and worked overtime, with one in three nurses working in the 24-h shift system. The growing number of infections among staff, as well as the need to quarantine following contact with an infected patient, resulted in difficulties in filling nursing shifts, meaning that the remaining group was obliged to work overtime shifts. The handling of the pandemic was aggravated by prior long-term negligence of the healthcare system in Poland; indeed, staff shortages existed even before the pandemic. The problem of nurse shortages was observed in 72% of Polish hospitals, and the average age of nurses in Poland is 53 years [22,27]. 

In our research, one-third of nurses pointed to a lack of proper procedures, training or a sufficient amount and availability of personal protective equipment. In everyday work, especially in the initial phase of the COVID-19 pandemic, Polish nurses faced many situations that intensified their feelings of uncertainty and fear. There was shortage of disinfection or self-protection resources. Additionally, organizational chaos, changes to regulations and treatment procedures for patients suspected of the infection aggravated negative emotions and hampered constructive control over them. Training sessions, clearly-defined procedures and work organization constitute crucial elements to prevent the escalation of negative emotions and stress [26]. Similar phenomena were observed by Chinese researchers [1,16]. In reality, no healthcare system, be it in Poland or elsewhere, was prepared for a situation such as the current COVID-19 pandemic. In the existing epidemiological situation, almost all of the nurses that participated in the present research felt that they could rely on support from coworkers, but only half on the support of their employers and supervisors. Only a quarter had access to psychological counselling. The protection of medical employees in the workplace during the SARS-CoV-2 pandemic ought to be a major priority. A particular focus on aid and support for mental health issues is necessary. It might be expected that nurses and other personnel directly engaged in the care and treatment of COVID-19-infected patients should be granted open access to counselling. Buchelt B. reported that 20% of studied nurses experienced the need to consult a psychologist [22]. Researchers from China demonstrated that the intensification of mental health problems was higher among medical personnel with limited access to psychological aid and education. They also showed that properly organized training sessions in hospitals helped employees to reduce stress levels, and that mental health counsellors employed by hospitals had the capacity to provide proper support [1,16,28]. Our research demonstrated that as many as three-quarters of nurses did not have access to professional aid from psychologists. The Chinese study, though not transferable to a Polish setting due to cultural, systemic and organizational differences, nonetheless constitutes a precious resource with the potential to inform decisions regarding actions to protect medical personnel working in pandemic conditions. The authors of that study point to the pivotal importance of psychological aid to prevent deviations compared to the treatment of deviations themselves among the already limited numbers of nursing staff. Although psychological counselling is offered in Poland by some institutions, and 24-h hotlines offering psychological consultations exist, there is still a shortage of resources such as of psychologists in the workplace despite a significant need, given the current circumstances [26,29].

### Study Limitations

The presented results are not without their limitations, which ought to be considered in future studies. Firstly, this study was performed on a small research group, and was limited only to one professional group. As such, future studies should include representatives of other medical professions like paramedics, physicians, medical carers, midwives and physiotherapists. The second limitation concerns the use of self-evaluation measures for anger, depression and fear. It has to be emphasized that while the collection of data via an internet poll provided information on the occurrence of behaviors indicating the suppression of negative emotions, i.e., anger, depression and fear, it was by no means equivalent to a formal psychiatric diagnosis. The third limitation concerns the lack of knowledge regarding the occurrence of mental disorders, especially depression, in the research group, because this would constitute an impeding factor for the research. 

## 5. Conclusions

The SARS-CoV-2 virus pandemic was found to have negatively affected the emotions of the surveyed nurses, most of whom noted having suppressed emotions including anxiety and depression as a coping mechanism. 

In the group of nurses diagnosed with COVID-19, the intensity of anxiety, suppression of depression and the perception of an increased workload were significantly higher.

The support from a psychologist and supervisors, including the possibility of undertaking SARS-Cov-2 tests in the workplace, are factors which could potentially alleviate the adverse mental states experienced by nurses.

A priority action in medical care facilities should be to launch psychological help, so that health problems can be prevented among the already limited nursing staff.

## Figures and Tables

**Table 1:** Description of the researched group.

		Group A (*n* = 79)		Group B (*n* = 79)		All Study Nurses (*n* = 158)	
		*n*	%	*n*	%	*n*	%
Gender, *n* (%)							
	Women	76	96.20	76	96.20	152	96.2
	Men	3	3.80	3	3.80	6	3.8
Age, *n* (%)							
	22–30 years	17	21.52	13	16.46	30	18.99
	31–40 years	22	27.85	28	35.44	50	31.65
	41–50 years	18	22.78	16	20.25	34	21.52
	51–66 years	15	18.99	21	26.58	36	22.78
	22–30 years	7	8.86	1	1.27	8	5.06
Marital status, *n* (%)							
	Married	44	55.70	44	55.70	88	55.7
	Single	22	27.85	19	24.05	41	25.95
	Informal relationship	11	13.92	14	17.72	25	15.82
	Divorced/Separated	2	2.53	2	2.53	4	2.53
Education, *n* (%)							
	Medical secondary education	10	12.66	8	10.13	18	11.39
	Bachelor’s degree in nursing	20	25.32	22	27.85	42	26.58
	Master’s degree in nursing	43	54.43	46	58.23	89	56.33
	Higher education, degree obtained in a faculty other than nursing	2	2.53	5	6.33	7	4.43
	Ph.D. degree	1	1.27	1	1.27	2	1.27
Seniority, *n* (%)							
	<year	1	1.27	3	3.80	4	2.53
	1–5 years’ work experience	28	35.44	29	36.71	57	36.08
	6–10 years’ work experience	7	8.86	12	15.19	19	12.03
	11–20 years’ work experience	15	18.99	11	13.92	26	16.46
	21–30 years’ work experience	16	20.25	18	22.78	34	21.52
	over 30 years’ work experience	12	15.19	6	7.59	18	11.39
Workplace, *n*(%)	Hospital—infectious diseases ward or 24-h outpatient care	28	33.44	26	32.91	54	34.17
	Hospital—noninfectious ward	51	64.56	53	67.09	104	65.83

Note: *n*—number; Group A—nurses diagnosed with COVID-19, Group B—healthy nurses who had never been infected with COVID-19.

**Table 2:** Frequency of symptoms of COVID-19 infection in Group A.

Main Symptoms Accompanying the SARS-CoV-2 Infection in the Researched Group	Group A *n* = 79	%
Asthenia	72	91.14%
Headache	61	77.22%
Loss of smell	57	72.15%
Muscle pain	55	69.62%
Loss of taste	49	62.03%
Cough	38	48.10%
Higher temperature	32	40.51%
Dyspnea	20	25.32%
Diarrhea	19	24.05%
Temperature above 38 °C	18	22.78%
No symptoms	1	1.27%

Note: The results do not add up to 100% due to multiple answers; Group A—nurses diagnosed with COVID-19.

**Table 3:** Correlation between COVID-19 infection and emotions experienced by nurses.

	Anger Suppression	Depression Suppression	Anxiety Suppression	General Coefficient of Emotion Control (CECS)	Anxiety Level Scale as Traits
	Mean	Mean	Mean	Mean	Mean
Group A	18.86	18.8	18.44	56.1	26.53
Group B	17.42	17.7	18.18	53.29	24.18
	*p* = 0.06	*p* = 0.11	*p* = 0.64	*p* = 0.08	*p* = 0.04 **

** Result statistically significant, Note: Group A—nurses diagnosed with COVID-19, Group B—healthy nurses who had never had COVID-19.

**Table 4:** Correlation between anxiety intensity according to the SL-C scale and emotional control level.

SL-C		Anger Suppression	Depression Suppression	Anxiety Suppression	General Coefficient of Emotion Control (CECS)
All study nurses	r	0.084	0.208	0.044	0.134
	*p*	0.29	0.009 **	0.7	0.09
Group A	r	0.137	0.288	0.081	0.194
	*p*	0.22	0.01 **	0.48	0.08
Group B	r	−0.017	0.086	−0.003	0.024
	*p*	0.88	0.45	0.97	0.86

** Result statistically significant; Note: r—Pearson’s correlation coefficient; *p*—significance level; Group A—nurses diagnosed with COVID-19, Group B—healthy nurses who have never had COVID-19.

**Table 5:** Correlation between COVID-19 infection among nurses and workplace conditions.

Working Conditions Workplace	All Study Nurses *n* = 158		Group A *n* = 79		Group B *n* = 79		Chi^2^ *p*
	*n*	%	*n*	%	*n*	%	
Hospital—noninfectious ward	104	65.83	51	64.55	53	67.08	ꭓ² = 11.065 *p* = 0.0008 **
Hospital—infectious diseases ward or 24-h outpatient care	54	34.17	28	33.45	26	32.82	
Performing work with patients suspected of being infected with the SARS-COV-2 virus							
All the time	57	36.08	30	37.97	27	34.18	ꭓ² = 3.824 *p* = 0.28
Yes, often or very often	56	35.44	26	32.91	30	37.97	
Yes, occasionally	42	26.58	23	29.11	19	24.05	
I have no contact with such patients	3	1.90	0	0.00	3	3.80	
COVID-19 procedures developed in the ward							
Yes	148	93.67	75	94.94	73	92.41	ꭓ² = 4.693 *p* = 0.09
No	6	3.80	4	5.06	2	2.53	
I don’t know	4	2.53	0	0.00	4	5.06	
Training for dealing with COVID-19 patients, as well as the use of protective clothing							
Yes	90	56.96	40	50.63	50	63.29	ꭓ² = 3.8224 *p* = 0.014 **
No, I had to learn everything myself	57	36.08	31	39.24	26	32.91	
I don’t know	11	6.96	8	10.13	3	3.80	
Sufficient provision of personal safety means in the ward							
Yes	110	69.62	56	70.89	54	68.35	ꭓ²= 0.029 *p*= 0.86
No	48	30.38	23	29.11	25	31.65	
Possibility of using personal safety resources without limitations							
Yes	111	70.25	55	69.62	56	70.89	ꭓ² = 0.0303 *p* = 0.86
No	47	29.75	24	30.38	23	29.11	
Possibility of undergoing a COVID-19 test at the workplace							
Yes, without problems	80	50.63	34	43.04	46	58.23	ꭓ² = 22.248 *p* = 0.00005 **
Yes, but I had to struggle for this	35	22.15	11	13.92	24	30.38	
No, because there were no swab points	16	10.13	11	13.92	5	6.33	
No, because I failed to obtain permission from my employer	27	17.09	23	29.11	4	5.06	
Nursing staff insufficiency							
Yes	133	84.18	71	89.87	62	78.48	ꭓ² = 6.307 *p* = 0.04 **
No	7	4.43	4	5.06	3	3.80	
Hard to evaluate	18	11.39	4	5.06	14	17.72	
Support from the employer							
Yes	90	56.96%	37	46.84%	53	67.09%	ꭓ² = 5.808 *p* = 0.02 **
No	68	43.04%	42	53.16%	26	32.91%	
Support from a psychologist in the workplace							
Yes	40	25.32%	12	15.19%	28	35.44%	ꭓ²= 7.531 *p* = 0.006 **
No	118	74.68%	67	84.81%	51	64.56%	
Support from coworkers							
Yes	152	96.20%	77	97.47%	75	94.94%	ꭓ² = 0.173 *p* = 0.67
No	6	3.80%	2	2.53%	4	5.06%	
A shortage of nursing staff in the workplace							
Yes	135	85.44	70	88.61	65	82.28	ꭓ² = 1.501 *p* = 0.47
No	19	12.03	7	8.86	12	15.19	
I don’t know	4	2.53	2	2.53	2	2.53	
Working overtime during the pandemic							
Yes	109	68.99	56	70.89	53	67.09	ꭓ² = 0.183 *p* = 0.73
No	49	31.01	23	29.11	26	32.91	
Number of work hours during the pandemic							
7.35 h shifts	18	11.39	8	10.13	10	12.66	ꭓ² = 3.665 *p* = 0.45
12 h shifts	91	57.59	44	55.70	47	59.49	
12 and 24 h shifts	26	16.46	14	17.72	12	15.19	
24 h shifts	21	13.29	13	16.46	8	10.13	
>24 h shifts	2	1.27	0	0.00	2	2.53	

** Result statistically significant. Note: n-number; ꭓ²—chi-square test; *p*—significance level; Group A—nurses diagnosed with COVID-19, Group B—healthy nurses who have never had COVID-19.
